# Coriander (*Coriandrum sativum* L.) from Alentejo (South Portugal)—Ethnobotany and Potential Industrial Use

**DOI:** 10.3390/foods13060929

**Published:** 2024-03-19

**Authors:** Orlanda Póvoa, Noémia Farinha, Violeta Lopes, Alexandra M. Machado, Ana Cristina Figueiredo

**Affiliations:** 1 VALORIZA—Centro de Investigação para a Valorização de Recursos Endógenos, Instituto Politécnico de Portalegre, Praça do Município 11, 7300-110 Portalegre, Portugal; 2 Banco Português de Germoplasma Vegetal (BPGV), Instituto Nacional de Investigação Agrária e Veterinária, Quinta de S. José, S. Pedro de Merelim, 4700-859 Braga, Portugal; violeta.lopes@iniav.pt; 3 Centro de Estudos do Ambiente e do Mar (CESAM Lisboa), Faculdade de Ciências da Universidade de Lisboa (FCUL), Biotecnologia Vegetal, DBV, C2, Campo Grande, 1749-016 Lisboa, Portugal; ialexam@gmail.com (A.M.M.); acsf@fc.ul.pt (A.C.F.)

**Keywords:** ethnobotany, traditional recipes, local markets, medicinal and aromatic plants (MAP), essential oils, fatty acids, extraction procedures

## Abstract

Coriander is a medicinal and aromatic plant (MAP) traditionally cultivated and used in Alentejo, Portugal. However, few publications are available about its ethnobotanical applications. Four independent ethnobotanical surveys were carried out: throughout the region (2002–2003), in three villages (2013), and in city markets (2007 and 2022). Coriander was the most common fresh cultivated MAP (75% of the total area) and also the most representative MAP fresh herb in city markets. The leaves, mostly, were used fresh or frozen or transformed in *piso*. Some of the recipes have agro-industrial potential, such as *piso* and aromatized olive oil. Coriander essential oils (EOs) were isolated by hydrodistillation from aerial parts with inflorescence emergence (APIs) and from fruits, and fatty acids (FA) by solvent extraction from the fruits. Gas chromatography and gas chromatography-mass spectrometry analysis showed EOs dominated by *n*-decanal (21–24%), 2-*trans*-decenal (12–18%) and *n*-nonane (10–17%) in APIs, and linalool dominance (73–78%) in the fruits. Petroselinic acid (32–55%) was the dominant fatty acid. A literature survey on conventional and nonconventional extraction techniques showed a constancy in the dominant compounds isolated, highlighted *piso* as a home-made green-extraction procedure, but also reflected the relevance of coriander as a MAP with diverse industrial potential uses.

## 1. Introduction

The term ethnobotany was introduced in 1895 by the American botanist John Harshberger and at the end of the 19th century, ethnobotany began to emerge as a science, offering a whole new field for pharmaceutical research [1,2,3]. Ethnobotany can be described in several contexts such as the study of the relationship between plants and humans since the term “ethno” refers to the study of people and “botany” to the investigation of plants [1,2,3].

Humans have used plants and natural products in general with a traditional understanding that dates back thousands of years. Ancient societies made extensive use of plants as spices or as the primary ingredients in food preparations. Plants were also the primary therapeutic agents used by humans until the middle of the 19th century, and their value in medicine is still acknowledged today as sources of bioactive compounds for the development of drugs and health-promoting products [1,2,3]. Recent studies focusing on the US market show that herbal dietary supplements sales surpassed USD 10 billion for the first time in 2020 [4,5,6,7].

The United Nations Sustainable Development Goals (UNSDG) represent a vision of a peaceful, fairer, and sustainable world. The third UNSDG is “Good Health and Well-Being” [8], which aims to enhance health by using health-enhancing plants and environmentally friendly practices in the food industry. Future dietary habits will be influenced by the advances made today since they may lead to the creation of plant-based goods with useful features that improve human health. The World Health Organization (WHO) reported that almost 80% of the global population depends on traditional medicine. WHO emphasized the significance of investigating medicinal plants for potential benefits to health (i.e., safety, efficacy, dose, clinical trials, toxicity, drug interaction, and therapeutic use). There is huge interest in aromatic plants as phytoconstituent-rich sources for nutraceuticals, innovative foods, and medications due to advancements in clinical nutrition. Due to their potential to improve health, interest in essential oils and plant-based active ingredients has grown [9,10].

The global demand for Medicinal and Aromatic Plants (MAPs) is on the rise, predominantly fueled by natural habitats as primary sources. In 2021, the European Union (EU) imported over 227 thousand tons of MAPs, with more than 50% originating from developing countries. Consequently, developing countries have emerged as pivotal suppliers of MAPs to Europe. From 2017 to 2021, the aggregate import volume of aromatic and MAPs into Europe exhibited an average annual growth rate of 3% [11,12].

*Coriandrum sativum* L. (coriander, family Umbelliferae or Apiaceae) is one of the oldest spices used also for its therapeutic properties. The origin of this MAP is the eastern Mediterranean and it’s grown in Europe, Africa, and Asia. All coriander parts are used as aromatic agents and/or as traditional medicines in different civilizations. Coriander leaves (cilantro or Chinese parsley) and coriander fruit (seed) are used in curry meat dishes, puddings, bread, soups, poultry, fish, and seafood dishes, or even to mask unpleasant odors in some foods. *C. sativum* contains high levels of vitamin B12, folate, vitamin C, vitamin A, and phenolics [2,9,13,14,15,16]. Due to its antimicrobial and antioxidant properties, coriander is used as an alternative food preservative [9,13,14,15].

In addition to coriander seasoning qualities, its nutritional and biological properties can be highlighted, including digestive, antioxidant, antimicrobial, hypoglycaemic, antihyperlipidemic, analgesic, anti-inflammatory and anticonvulsant activities, among others. These properties are due to the presence of several classes of phytoconstituents like carotenoids, tannins, phenolic acids, flavonoids, coumarins and terpenes [3,9,17,18,19,20]. Coriander roots and seeds contain bioactive phytochemicals (i.e., gallic acid, thymol, and bornyl acetate). Linalool is the main leaf volatile constituent responsible for several coriander therapeutic traits. Other active ingredients in coriander include essential oils, fatty acids, tocols, and sterols. Varieties, genotypes, ecotypes, planting season and condition, plant part, growth stage, harvesting period, and extraction method all affect the yields and compositions of these components. Both coriander aerial parts and seeds provide solvent extracts, water-soluble compounds, fixed oils, and essential oils. Coriander essential oil ranks second highest in the global annual production [9].

Coriander benefits can be extended to related fields, such as the cosmetics, agricultural and food industries. Due to its everyday use and medicinal properties, coriander is a highly interesting functional food [16,18].

Located at the south of the Tagus River, Alentejo is one of the seven territorial units of Portugal. It is a territory of particular interest for its morphology, flourishing vegetation, and history, having been a meeting area among different civilizations, Phoenicians, Celts, Greeks, Romans, Arabs, Jews and Christians. *Açorda* traditional recipes were a gift from the presence of the Arabs in Alentejo’ lands.

Coriander is used as a folk plant in the south of the Tagus River, where is one of the most popular and extensively grown aromatic and food seasoning species, giving a distinctive flavor to this region’s traditional cuisine. Most commonly, Portuguese from the North of the Tagus River, dislike its taste and aroma. Coriander is not commonly used in recipes in the northern areas of Portugal, such as Beira Baixa and Beira Litoral, where it is used in just 2.8% and 2.1% of recipes, respectively. On the other hand, in the southern region of Portugal, coriander leaves are used in 8.6% of Estremadura recipes, 13.4% of Algarve recipes, 14.3% of Ribatejo recipes, and 18.4% of Alentejo recipes [21]. Nowadays, the use of coriander in food seasoning is also recommended to reduce salt [22].

At the end of the 20th century and the first decade of the 21st century, ethnobotanical studies were carried out in line with the dynamics of MAP research in the Iberian Peninsula [1,23,24,25,26,27,28]. Alentejo is characterized by an ethnic plurality and a rich ethnobotanical knowledge, although the available information is fragmentary, widely dispersed, and often guarded in oral popular culture.

The main goal of this study was to compile the findings from multiple initiatives carried out at the Escola Superior Agrária de Elvas/Instituto Politécnico de Portalegre (ESAE/IPP), with an emphasis on the agronomic features of the most widely utilized species as well as the gathering and preservation of traditional Alentejo recipes. To identify MAP species of commercial relevance, numerous surveys were conducted at both indoor and outdoor marketplaces, and interviews were conducted with local MAP producers. Notably, coriander emerged as a significant MAP plant. The ethnobotanical data was complemented with other significant qualities, such as the essential oil profile and total fatty acid composition, in addition to a survey on the advancements in coriander extraction technologies, with the goal of locally potentiating improved coriander varieties.

## 2. Materials and Methods

### 2.1. Ethnobotanical Surveys: Food Seasoning with Coriander in Alentejo Region

#### 2.1.1. Surveys Conducted in 2002 and 2003

**Figure 1:**
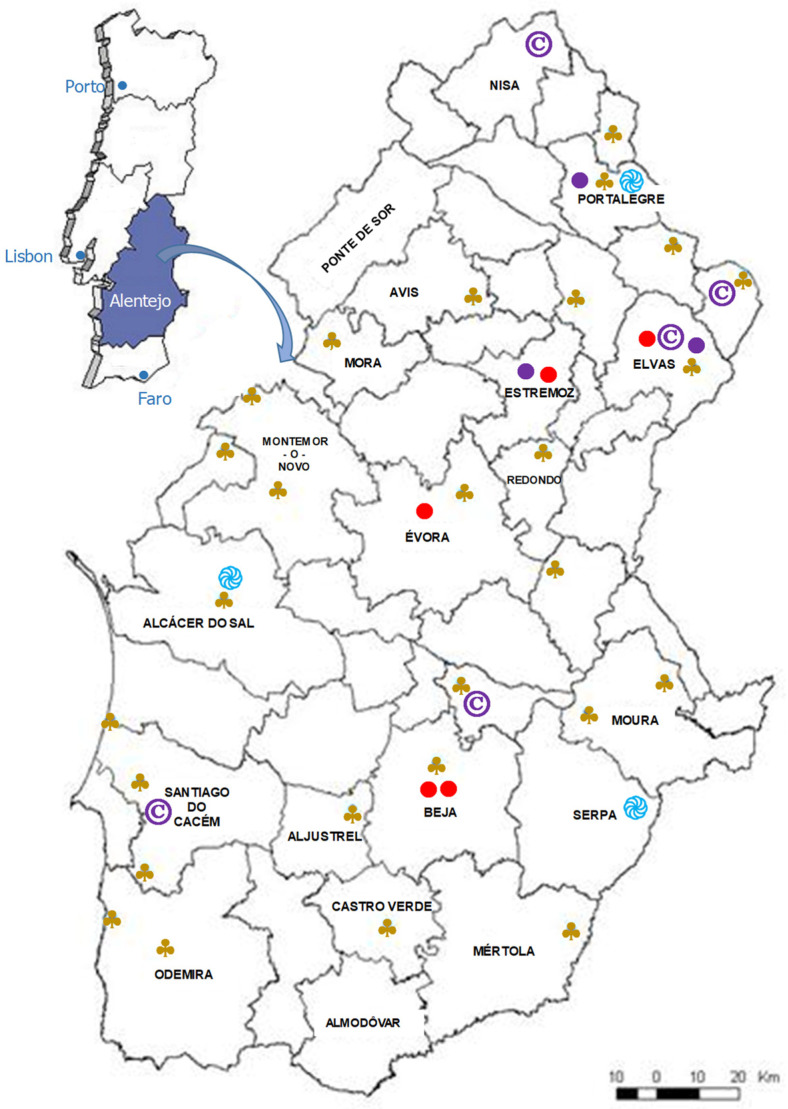
Ethnobotanical surveys in the Alentejo region during 2002–2003 (♣) and 2013 (֎). Mainland Portugal in the top left corner with Alentejo region evidenced in blue. Arrow indicates detail of Alentejo municipalities. Visited markets in 2007 (●) and 2022 (●). Geographic origin of coriander landraces used in chemical characterization analysis (©).

During 2002 and 2003, 28 semi-structured interviews were carried out throughout the Alentejo region (Figure 1), with the goal of collecting coriander regional landraces and data on its ethnobotanical uses [29,30,31]. The survey questionnaire, included in Supplementary Materials Table S1, was carried out within project Agro 34 [32].

#### 2.1.2. Surveys Conducted in 2013

The ethnobotanical survey conducted in 2013, benefiting from the experience of the previous surveys, focused on gathering the frequency of use of eight categories of food seasoning recipes [*açorda*, Fabaceae (beans, chickpea, pea), fish, fish soup, meat, *migas*, *piso* and rice]. Concrete food recipes with coriander were also registered. The surveys were carried out in three Alentejo villages, selected to calculate the representativeness of coriander use in a population of known size. The three villages were chosen based on their location in three different subregions of Alentejo.

Considering the 2011 population census [33], a total of 24 interviews (1.4% of the inhabitants) were carried out in Alegrete (North Alentejo), 20 interviews (1.2% of the inhabitants) in Santa Catarina (Alentejo Litoral) and 21 interviews (2.2% of the inhabitants) in Vale de Vargo (Baixo Alentejo) (Figure 1), making an average of 1.6% inhabitants interviewed in the 3 villages. Since each household was only surveyed once, the actual representation was higher given that the typical family size in the area is larger than one member. The survey questionnaire is included in Supplementary Materials Table S2.

The plants mentioned in the recorded recipes and recipe citations, were botanically identified. The species employed in the recipes were divided into three categories, large, medium, and small amounts, according to the used quantities.

### 2.2. Ethnobotanical Surveys: Coriander Representativeness on Local Markets

The Alentejo region is divided into three subregions: Alto Alentejo, whose administrative center is the city of Portalegre; Central Alentejo, whose administrative center is the city of Évora; and Baixo Alentejo, whose administrative center is the city of Beja.

Every subregion’s administrative center was included in the surveys of city markets: Portalegre (2022 surveys), Évora (2007 surveys) and Beja (2007 surveys). Furthermore, 2 important cities in the region were included in the survey collection: Elvas, in 2007 and 2022 surveys, and Estremoz in 2007 and 2022 surveys, Figure 1. The data collection in the cities markets, both in 2007 and 2022 surveys, included all of the MAP sellers present in the marketplaces.

#### 2.2.1. Surveys Conducted in 2007

From December 2006 to March 2007, 5 markets in the Alentejo region were visited, interviewing 33 MAP producers in the indoor markets of Beja (8 producers), Elvas (2 producers), and Évora (4 producers), and in the outdoor markets of Beja (9 producers) and Estremoz (10 producers). 

This study gathered information on MAP with commercial interest (list of MAPs sold) and species agronomic features (cultivated area for each MAP), such as the number of sowing dates per year, number of plants cuts per year, provenance of the seed (landrace or acquired), the importance of obtaining new varieties and the most important features for a new variety. The survey was included within Project Agro 522 [34]; the questionnaire is included in Supplementary Materials Table S3.

#### 2.2.2. Surveys Conducted in 2022

The 2022 surveys were carried out to continue assessing the information about the MAP species with commercial interest in the Alentejo region markets [35]. The survey took place in the markets of Elvas, Portalegre and Estremoz, based on two visits (autumn 2020 and spring 2022). The survey included recorded data regarding sellers of dried wild plants and imported spices; however, since coriander is a cultivated species, always sold fresh, only the information pertaining to sellers of fresh vegetables was included in this study to facilitate comparisons with surveys conducted in 2007. Thus, a total of 28 MAP sellers (4 from Elvas, 11 from Portalegre and 13 from Estremoz) were considered. Two localities are shared by the 15-year interval local market surveys.

The list of MAP sold, the origin of the plants (cultivated or wild), the type of plant (fresh, dried, potted) and the packaging (bulk, plastic, paper, other) were recorded. The survey questionnaire is included in Supplementary Materials Table S4.

### 2.3. Chemical Characterization of Coriander Accessions

The chemical characterization of selected accessions was performed by evaluating their essential oils (EOs) and total fatty acid composition, Table 1.

Except for samples obtained from the wild collection, the remaining *C. sativum* plant materials were obtained from the experimental fields of Escola Superior Agrária de Elvas/Instituto Politécnico de Portalegre (ESAE/IPP). Representative material was deposited at the Banco Português de Germoplasma Vegetal (BPGV) under each own accession number. The plant material was obtained for two consecutive years (2013 and 2014). The sampling details for each of the analyzed plant materials, and their origin, are provided in Table 1, and detailed in Supplementary Material Figure S1.

The aerial parts of the coriander with inflorescence emergence were collected in the agronomic essay and transported in paper bags, inside a thermal box, directly from the field to the FCUL laboratory. The coriander fruits were obtained from the agronomic trial field of ESAE/IPP, sown on 30 November 2014, with harvesting in June 2015, when they were fully ripe. The samples were transported in paper bags to the laboratory. Subsequently, they were mechanically threshed and stored in paper bags at room temperature until they were sent to the chemical analysis laboratory at FCUL on 30 November 2015.

#### 2.3.1. Essential Oils Isolation and Analysis

The EOs of 11 samples were obtained by hydrodistillation (HD), using a Clevenger apparatus [36], and analyzed by Gas Chromatography (GC) and Gas Chromatography-Mass Spectrometry (GC-MS) for component quantification and identification [37,38,39,40,41,42,43,44,45,46,47], respectively, according to Machado et al. [48], as fully detailed in Supplementary Material Section S2.3.

#### 2.3.2. Fatty Acids Isolation and Analysis

Total fatty acids of 17 samples were isolated, using a Soxhlet apparatus. After transesterification to fatty acid methyl esters (FAMES), GC and GC-MS analyses were run as fully detailed in Supplementary Material Section S2.3. Except for the fruits obtained from the wild plants (Cs_31_F_14_w and Cs_33_F_14_w), the remaining data on total fatty acids is presented as an average of each three samples obtained for each plant material, Table 1.

### 2.4. Numeric Data Statistical Treatment

The data statistical treatment of all of the ethnobotanical surveys was carried out using Excel [49] spreadsheets, with the creation of lists of cited species and calculations of citation frequencies.

The relationship between the various samples was evaluated using the EOs’ and fatty acids percent composition, through cluster analysis using the Numerical Taxonomy Multivariate Analysis System (NTSYS PC software, version 2.2, Exeter Software, Exeter University, Exeter, UK) [50]. The sequential agglomerative hierarchical nested cluster analysis was chosen as the agglomerative clustering method. The effect of different scales of identification was eliminated by standardizing the percent composition data matrix. The correlation coefficient was chosen as the measure of sample similarity for the cluster analysis, and the cluster definition was carried out using the unweighted pair group method with arithmetical averages. The correlation degree was assessed according to Pestana and Gageiro [51] as very high [0.90, 1.00], high [0.70, 0.90[, moderate [0.40, 0.70[, low [0.20, 0.40[, and very low (<0.20).

## 3. Results and Discussion

### 3.1. Ethnobotanical Surveys: Food Seasoning with Coriander in Alentejo Region

#### 3.1.1. Surveys Conducted in 2002–2003

During this survey, 62 coriander recipes or recipe names expressively naming plants were recorded, including 23 complete recipes. Among popular recipes three traditional ones stood out, *açorda*, a traditional Alentejo bread soup with fish, or codfish, and egg, seasoned with coriander, pennyroyal (*Mentha pulegium* L.) or hart’s pennyroyal (*M. cervina* L.), *migas*, a meat side dish based on Alentejo bread seasoned with coriander, and *piso*, a more fluid or spread preparation, made from coriander, pennyroyal or hart´s pennyroyal, garlic, olive oil and salt.

Most people used coriander more than once a week, an extremely common usage. Primarily the leaves, but also the tender stems were used fresh or frozen or transformed in *piso* similarly to pennyroyal [27]. The most popular recipes were *açorda* (traditional bread recipe) and fish, but with a broad utilization on salads, omelets, meats, infusions, etc. [31].

#### 3.1.2. Surveys Conducted in 2013

Based on the 2013 ethnobotany survey, the four most important coriander recipe categories were: *açorda*, fish, rice, and Fabaceae (mostly beans) (Table 2). A total of 63 citations of coriander usage as a spice, including 51 complete recipes were recorded.

The average representativeness of these results was 1.6% (considering the number of inhabitants interviewed in the total population of the villages in the 2011 census).

#### 3.1.3. Surveys Conducted in 2002–2003 and 2013

This section includes the results of the concrete examples of coriander recipes from the 2013 surveys, with descriptive text (part 7 of the survey, Table S2), as well as the recipe descriptions from the 2002–2003 surveys. The studies considered a total of 125 recipes or recipe names in all; of these, the majority (36) were recipes of beans and other Fabaceae, followed by *açorda* (29) (Table 3).

**Figure 2:**
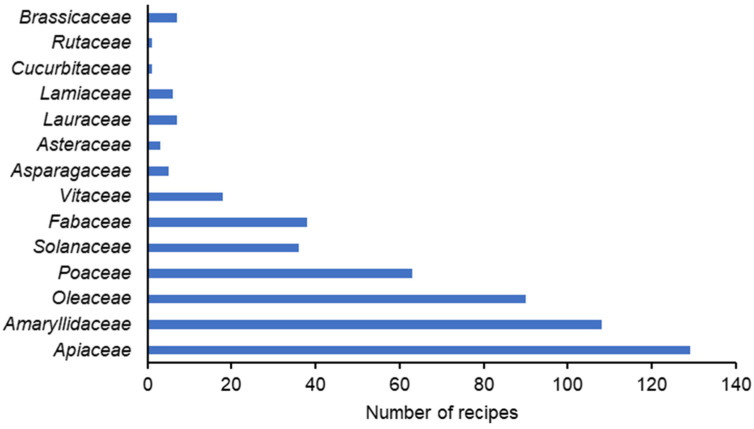
Representativeness of plant species botanic families on coriander traditional recipes. 2002–2003 and 2013 surveys.

Plants from 14 botanic families were mentioned. The most cited botanic families were Apiaceae (coriander), Amaryllidaceae (garlic, onion), Oleaceae (olive), Poaceae (wheat, rye, corn, rice), Fabaceae (bean, brad bean, chickpea), Solanaceae (potatoes) (Figure 2).

**Figure 3:**
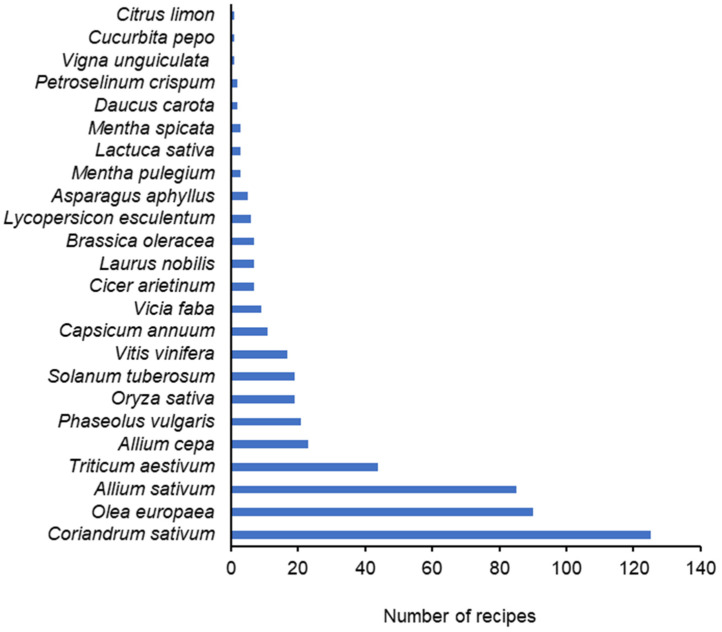
Representativeness of plant species on coriander traditional recipes. 2002–2003 and 2013 surveys.

Taking into account the results of both surveys (2002–2003 and 2013), 512 references to plant species were found in the recipes; these references accounted to 24 plant species, belonging to 14 botanical families (Figure 3). Twelve species, as *Triticum aestivum* L. (wheat, in bread and flour), *Orysa sativa* L. (rice), *Solanum tuberosum* L. (potato) and *Phaseolus vulgaris* L. (bean) were consumed in large amounts, being the most representative in the recipes. Four plant species were consumed in average amounts, including *Allium cepa* L. (onion) and *Olea europaea* L. (olive, as olive oil and olives). Spices were consumed in small amounts, of which the most representative were *Coriandrum sativum* L. (coriander), *Allium sativum* L. (garlic), *Vitis vinifera* L. (vine, vinegar) and *Mentha pulegium* L. (pennyroyal) (Figure 3). 

These surveys were also valuable for assessing the genetic erosion of the species [29] and the collection of germplasm to continue the ESAE/IPP coriander plant breeding program [30].

The survey’s results obtained in 2013 were like those from 2002–2003 regarding the main families and species used in traditional coriander recipes, supporting the key findings from Póvoa et al. [27,31]. *Açorda* (with *Triticum aestivum* bread) appears as the most cited recipe, both in 2002/2003 surveys and 2013 surveys. Considering the recipes with plants as major constituents, coriander rice (*Oryza sativa*), stewed beans (*Phaseolus vulgaris*), stewed chickpea (*Cicer arietinum*) and stewed broad bean (*Vicia faba*) were also very frequently mentioned, in both surveys, which confirms the Mediterranean nature of this cuisine.

### 3.2. Coriander Representativeness on Local Markets

#### 3.2.1. Surveys Conducted in 2007

**Figure 4:**
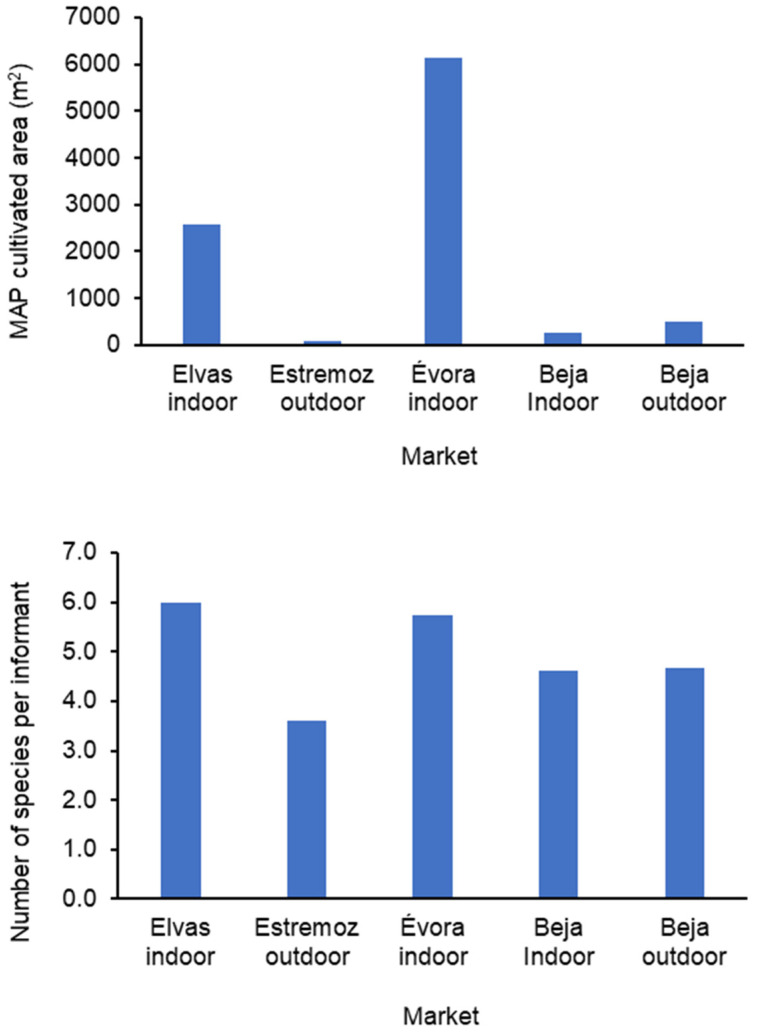
Cultivated area (**up**) and number of plant species (**bottom**) from MAP producers on Elvas, Estremoz, Évora and Beja markets (2007 surveys).

The MAP producers from Évora indoor market had the highest average area dedicated to MAP production (about 6000 m^2^), followed by Elvas indoor market (about 2500 m^2^). Most of the remnant producers cultivated their MAP on small plots (less than 1000 m^2^). The number of sold MAP species by producer varied from 3 to 6 (Figure 4). 

**Figure 5:**
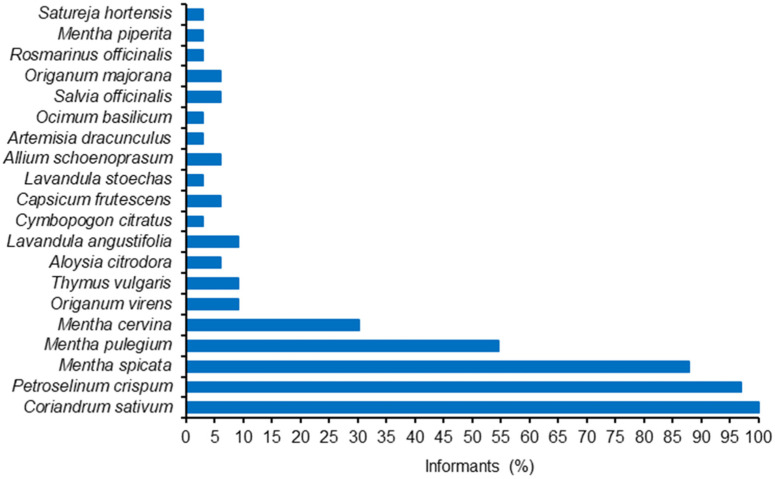
Cultivated MAP species and % informants who cultivate each species (2007 surveys) according to MAP producers in Elvas, Estremoz, Évora and Beja markets.

**Figure 6:**
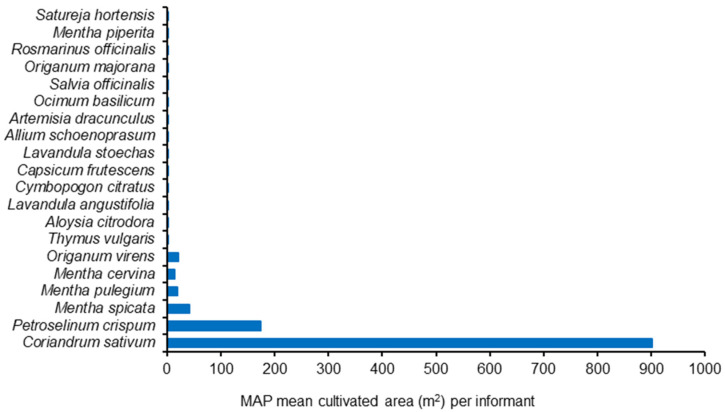
Mean cultivated area (m^2^) per informant for each MAP species (2007 surveys) according to MAP producers in Elvas, Estremoz, Évora and Beja markets.

**Figure 7:**
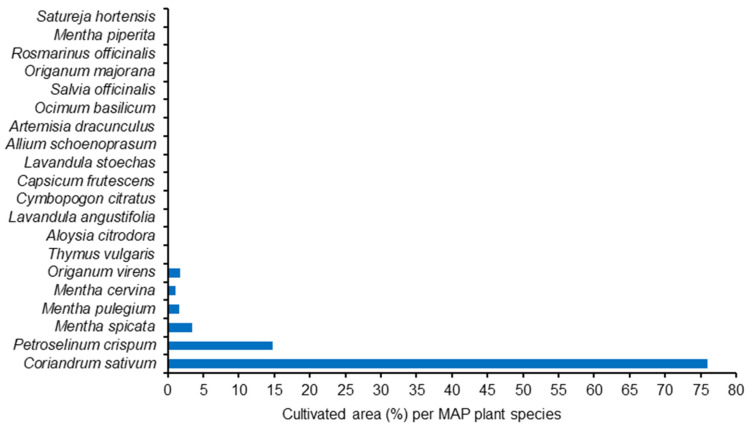
Cultivated area (%) for each MAP species (2007 surveys) according to MAP producers in Elvas, Estremoz, Évora and Beja markets.

All of the interviewed MAP producers cultivated coriander (Figure 5). Parsley (*Petroselinum crispum* (Mill.) (Mill.) Fuss) and mint (*Mentha spicata* L.) were also very common cultivated species, but with lesser incidence. Considering the percentage of total MAP cultivated area, coriander clearly dominates (about 75%), which coincides with the official statistics from the Portuguese government [52] giving 71% of the MAP cultivated area to coriander (Figure 6 and Figure 7).

**Figure 8:**
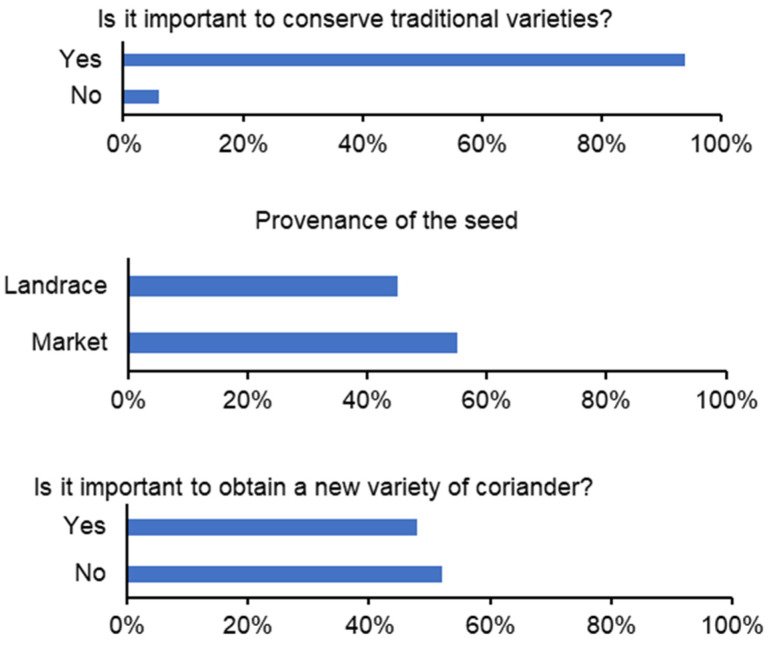
The importance of traditional varieties conservation (**top**), provenance of the seeds (**middle**) and the importance of the development of new varieties (**bottom**) (2007 surveys) according to MAP producers in Elvas, Estremoz, Évora and Beja markets.

**Figure 9:**
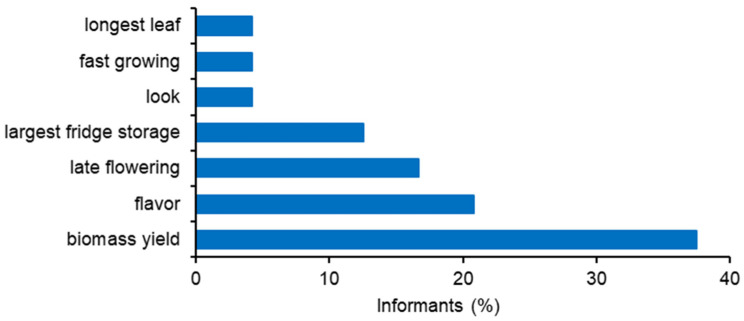
Most important coriander features for plant breeding programs (2007 surveys), according to MAP producers in Elvas, Estremoz, Évora and Beja markets.

Most of the interviewed MAP growers considered that it was important to have a coriander traditional varieties (landraces) conservation program, however, most cultivate using purchased seeds. The informants didn’t recognize the importance of developing new coriander varieties (52%) (Figure 8). The most important features for coriander plant breeding programs were biomass yield, flavor, and late flowering (Figure 9).

#### 3.2.2. Surveys Conducted in 2022

Four sellers were found in the Elvas market, which sold 11 fresh MAP species, 16 dry species, of which 10 from the wild collection. In the Portalegre market there were 11 vendors of 17 fresh MAP species, 35 dried species, of which 23 were from the wild. In the Estremoz market there were 13 sellers of 33 fresh MAP species in bulk or potted (in nursery plant containers), 75 dried species, of which 31 were from the wild. 

The cultivated MAP species represented 66% of the total marketed species, while wild collected species represented 34%. A total of 69 MAPs collected from the wild were identified in the 3 markets (unpublished results from the authors). 

**Figure 10:**
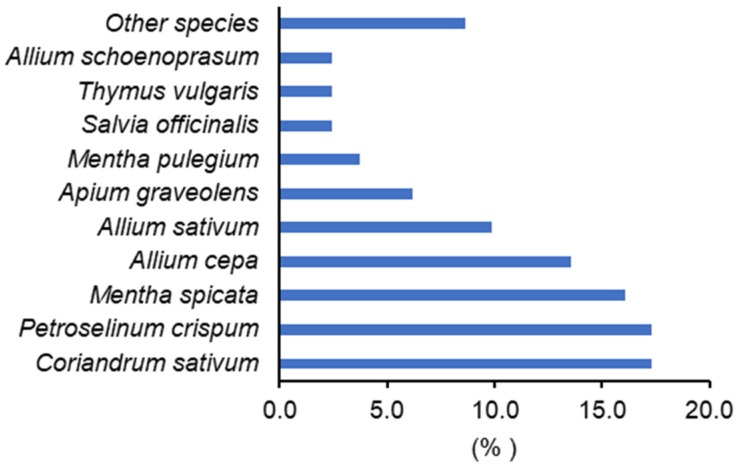
Representativeness of the fresh cultivated medicinal and aromatic plants (MAP) in the markets of Elvas, Estremoz and Portalegre (2022 surveys).

Most cultivated MAPs were marketed fresh (54%), in bulk (44%), potted (35%) or in plastic bags (28%). Considering the cultivated fresh MAP sector, most of the plants were sold in bulk (97.5%), with a total of 17 species. Coriander (*Coriandrum sativum*) represented 17% of the observed species, parsley (*Petroselinum crispum* (Mill.) Nyman ex A. W. Hill) with 17% and spearmint (*Mentha spicata* L.) with 16% were also very frequent (Figure 10).

There were species simultaneously on the list of cultivated and wild species, such as laurel (*Laurus nobilis* L.), linden (*Tilia platyphyllos* Scop.), pennyroyal (*Mentha pulegium* L.), and oregano (*Origanum vulgare* spp. *virens* Hoffmanns. et Link.). In addition to the wild harvest, they were also grown for sale in bulk or in pots (unpublished results from the authors).

The survey should be carried out in other marketplaces to complete the list of commercialized MAPs.

#### 3.2.3. Surveys Conducted in 2007 and 2022

Coriander appears in the 2 ethnobotanic surveys as the most representative MAP species in the Alentejo region markets, concerning the fresh plant sector, which justified the continuity of the plant breeding program developed at ESAE/IPP.

### 3.3. Coriander Aerial Parts and Fruits Essential Oils

The yields of the EOs isolated from *C. sativum* aerial parts with inflorescence emergence (APIs) ranged from 0.04% to 0.05% (*v*/*w*), whereas the fruits EOs yields ranged from 0.1% to 0.5%, Table 1.

There were fifty-five compounds identified in the APIs EOs and forty-four in the fruits EOs, for a total identification percentage >94%. Table 4 lists the relative amounts of all of the identified compounds on each EO, with their elution order in the DB-1 column.

*n*-Decanal (21–24%), 2-*trans*-decenal (12–18%), *n*-nonane (10–17%), and 2-*trans*-decen-1-ol (8–10%) were the dominant components (≥10%) of APIs EOs. The fruits EOs were characterized by linalool dominance (73–78%), followed, in much lower amounts, by α-pinene (3–9%) and γ-terpinene (4–8%). 

As detailed in Figures S2 and S3, agglomerative cluster analysis, based on the chemical composition of all coriander EOs analyzed, corroborated the qualitative and quantitative differences between APIs and fruits EOs. Interestingly, for both the APIs and the fruits EOs, there was some consistency in the observed groupings. With a very high degree of correlation, CS_1 paired with Cs_TP both in API and fruits EOs, as well as Cs_16 with Cs_32. In the fruits EOs, Cs_31_F_14 paired with that from wild collection, Cs_31_F_14_w, Figure S1.

The yields, as well as the general composition of both API and fruits EOs agree with what was reported for this species EOs, isolated both from plants grown in Portugal or in other countries, as described by Machado et al. [48]. Most commonly, coriander aerial parts EOs are dominated, in variable amounts, by 2-*trans*-decenal, *n*-decanal, 2-*trans*-dodecenal, 2-*trans*-decen-1-ol, among other components as detailed in references in Machado et al. [48]. More consistently, the fruits EOs are dominated by linalool, most published data being within ISO specifications (65–78%) [48,53].

### 3.4. Coriander Fruits Fatty Acids

The yields of the fatty acids isolated from *C. sativum* fruits ranged from 0.6% to 1.3% (*w*/*w*), Table 5. 

Thirteen fatty acids were identified in the fruit’s extracts, with a total percentage of identification >93%. The relative amounts of all fatty acids identified are listed in Table 5, following their elution order in the DB-1 column.

Petroselinic acid (32–55%) was the dominant fatty acid in coriander extracts, followed by palmitic acid (12–30%), Table 5. Despite the high correlation (Scorr > 0.84), hierarchical clustering, based on the fatty acid composition showed sample Cs_16 separated from the remaining six, even more correlated samples (Scorr > 0.92), Figures S4 and S5, due to the lower amount of petroselinic acid (32%). This clustering is also supported by the relation between fatty acids ratios in Table 5.

The European Commission (EU) decided to approve coriander seed oil as a novel food ingredient in food supplements in 2014, with a maximum daily intake of 600 mg [54]. This decision specifies a petroselinic acid content of 60–75%, followed by linoleic (12–19%), oleic acid (8–15%) and palmitic acid (2–5%). 

According to Uitterhaegen et al. [55] petroselinic acid may represent 31–75% of the fatty acid profile. These authors compared the quality of fatty acid composition of coriander Soxhlet extracted oil and twin-pressed oil, and they found an average value of 73% of petroselinic acid with both extraction procedures, which is consistent with previous reported data [56,57].

The petroselinic acid percentages obtained in the present study were within the lowest range reported in the literature [55,56,57], and somewhat below EU specifications [54], namely in oleic acid content which was found only in trace amounts. This may be due to diverse operative conditions pre-extraction (with, or without griding, for instance), in extraction type and analysis (without, or with solvent and temperature, and or solvent type, and extraction time), or other factors, such as the ripening stage of the fruits, or coriander variety. In addition, the analytical chromatographic procedure can also induce some variations, as in some studies instead of FAMES analysis, derivatizing agents were used. Thus, it would be important to test for each coriander variety, the effects of the fruit’s maturity state, the significance of grinding the fruit, or not, before extraction, and the various types of extraction, such as cold-pressed, without or with solvent, extraction with hot solvent, extraction with different types of solvent, time of extraction, and diverse analytical conditions in order to better respond to and evaluate the reason for these differences.

### 3.5. Advances in Coriander Extraction Methods

The relevance of coriander on itself (fresh or dry, whole, or ground), and of its bioactive rich extracts, is supported on several dedicated standards and monographs [53,58,59,60,61]. In addition, coriander is an interesting plant species as being an example of the same plant providing very different bioactive compounds according to the plant part used for extraction (aerial parts or fruits, also named seeds), or, for the same plant part (fruits), according to the methodology of extraction. 

An ideal extraction procedure would be highly efficient, rapid, easy to apply, sustainable, and non-destructive. Nowadays, there is a wide range of extraction techniques for recovering diverse bioactive compounds, which fall into two categories, conventional and nonconventional techniques, with their corresponding advantages and disadvantages. The former include maceration, soaking, Soxhlet extraction, water percolation, solid–liquid extraction with organic solvents, or distillation procedures. These techniques require a considerable extraction time, with the possibility of thermal degradation of thermolabile bioactive compounds or they use organic solvents such as *n*-hexane, which despite allowing good recovery of lipid compounds, have some drawbacks, such as toxicity to humans and the environment [62,63,64,65]. Therefore, in recent years several nonconventional techniques have been explored to minimize these limitations, such as microwave-assisted extraction, ultrasound-assisted extraction, pulsed electric field extraction, ohmic heating, enzyme digestion, subcritical water extraction, supercritical carbon dioxide extraction, or the use of natural deep eutectic solvents (NADES) [64,66,67].

Despite the existence of other types of extracts, coriander is mostly known for its essential oil (EO) obtained from either the aerial parts or fruits, and for its vegetable oil (oil), extracted from the fruits. In both cases, these extracts result from different, but conventional, techniques. An EO can only be isolated by one of two procedures, by either steam-, hydro- or dry- distillation of any plant part, or, from the epicarp of citrus fruits, by a mechanical process without heating, designated cold-pressing. The main advantage of obtaining an EO by distillation is using only water as solvent, although at high temperatures. A vegetable oil is either obtained by solvent extraction or by mechanical pressing, without, or with heating. These two types of extracts have very different compositions, and thus also diverse biological properties. For this reason, EO and oil should not be used as synonyms, nor should these designations be attributed to other types of extracts. Another advantage of both the EO and oil from coriander is that their main compositions are well established internationally, thus allowing the assessment of their quality [53,54].

Aroma is a fundamental food characteristic that is decisive in consumer perception and acceptance. *C. sativum* is an aromatic herb frequently used as a food additive and flavor enhancer, as a spice and condiment. In addition to culinary applications, coriander is also used in traditional medicine, cosmetics, and the food industry [62,68,69]. The characteristic aroma and pleasant flavor of coriander is due to volatile components isolated from the seeds (fruit), or from the aerial parts, as an EO. The fruits EO, with the oxygen-containing monoterpene, linalool as the main component, is applied in medicine as a carminative or as a flavoring agent to mask the bitter taste of other preparations [70,71]. Despite the aerial parts of coriander not being as well studied as the seeds, aliphatic aldehydes, and alcohols, such as *trans*-2-decenal, decanal, *trans*-2-dodecenal and 2-decen-1-ol, were the dominant compounds in the fresh herb EO, responsible for the characteristic aerial parts’ odor [68,69].

Although in lower amounts than other oil-rich fruits or seeds, the lipid components of coriander fruits (seeds) are thought to contribute to the development of distinctive aromas and flavors during ripening, since they are regarded as the precursors of several of the fruit’s volatile odor principles. Thus, fatty acids, sterols, and tocols can be found in the seeds lipidic fraction (oil), with petroselinic acid as the major fatty acid identified followed by linoleic acid. Identification of the fatty acids in coriander leaves indicated a dominance of polyunsaturated fatty acids, with α-linolenic as predominant, followed by linoleic, heptadecenoic and palmitic acids [14].

In addition to essential oils (terpenes rich), and oil (fatty acids rich), by using other extraction procedures, different compounds can be isolated from coriander seeds or aerial parts (leaves and stems). Condensed tannins and flavonoids were identified in the methanolic seed extract, and flavonoids, phenolic acids and polyphenols were found in the leaves and stems [3,72].

Although not providing an EO, subcritical water extraction was compared with hydrodistillation and Soxhlet extraction in volatile oil isolation from the seeds, with the former yielding a volatile oil that was more concentrated in valuable oxygenated components, despite the lower extraction efficiency. This technique is based on the use of water, at temperatures between 100 °C and 374 °C and at a pressure high enough to maintain the liquid state. With this technique, polar compounds are extracted at lower extraction temperatures of water and nonpolar at higher extraction temperatures. Therefore, by controlling the polarity of the solvent by adjusting the temperature, terpenes may be extracted selectively according to their structural properties [62,70,73]. Song and Ko [73] also reported that subcritical water extraction has reduced the time necessary for extraction compared to methanol, hexane, hot water and hydrodistillation, making it an applicable technique for extracting terpenes from seeds. This method’s main advantages over conventional extraction procedures include shorter extraction times, higher-quality extracts, less extraction agent costs, and being an environmentally friendly technique [70,74]. 

Supercritical fluid extraction was another extraction technique studied as an alternative method to hydrodistillation and steam distillation to overcome hydrolysis and thermal degradation in coriander seeds. Studies reported that the volatile oil obtained by supercritical CO_2_ extraction provided a higher yield when compared to the other techniques used, namely Soxhlet-dynamic headspace, solvent extraction, steam distillation and hydrodistillation. Linalool represented the main compound, regardless of the extraction procedure used, as opposed to the other components which differed in accordance with the isolation technique. These findings revealed a marked influence of the method applied on the composition of the minor compounds [71]. Abbas et al. [75] reported coriander aerial parts essential oil yield isolated by hydrodistillation was a little higher than the extract obtained by supercritical fluid extraction. However, when compared to the essential oil obtained by hydrodistillation, the extract obtained by supercritical fluid extraction had a higher biological potential due to the presence of a larger number of bioactive compounds.

The microwave assisted hydrodistillation method is another technique that can be used to obtain volatile oil from coriander seeds. This method is recognized as environmentally friendly given its shorter extraction time, reduced energy consumption, which results in lower costs, environmental compliance, improved heat flow and lower solvent consumption. Briefly, the microwave waves are converted into thermal energy, which leads to the heating of the solvent and the sample in the microwave oven, resulting in a faster extraction process. A comparative study on the extraction of essential oil from coriander seeds performed by hydrodistillation and microwave-assisted hydrodistillation showed that the yield was very similar between both processes, 0.31% and 0.33%, respectively. Most of the identified compounds were found in both extracts, with linalool as the dominant one. It was also noticed that microwave-assisted hydrodistillation gave the highest oxygenated monoterpenes content, although the number of identified compounds was lower [64]. 

As an example of a non-invasive technique, headspace-solid phase microextraction was used to characterize the volatiles present in coriander aerial parts. Among the various compounds, the content of aldehydes was dominant, mostly represented by *trans*-2-tetratecenal, followed by alcohols, hydrocarbons, and esters [76].

Scandar et al. [67], used several natural deep eutectic solvents (NADES) as additives in coriander hydrodistillation. The use of choline chloride (ChCl)-urea enhanced the extract yield compared to the other tested NADES and to water. There were not many variations among extracts composition (except for those obtained with the NADES acidic ChCl-citric acid, which evidenced a possible impact on the final extract composition).

Interestingly, some common recipes can be considered green-extraction procedures, and methods of preserving the obtained product. This is true for the *piso*, one of the most recorded recipes in this study. In *piso*, olive oil serves as the green-solvent in a home-made vegetable oil-extraction of coriander active principles. Consumers can directly use this extract as an aromatized olive oil or spread.

The potential applications of coriander could target different areas, most notably its fruits essential oil, with preservative properties due to the antibacterial activity of its compounds, with environmentally friendly features as well as being a biodegradable agent suitable for food preservation. Coriander essential oil has also been recognized as a safe flavoring and/or preservative ingredient in food products, by the Food and Drug Administration [2,77].

Given the aforementioned biological properties of its constituents, coriander could also be considered a promising functional food contributing to the promotion of healthy living, in the actual context of ageing and lifestyle-related diseases [16]. The lipidic fraction obtained from the seeds can also be applied in different industries, such as the plastic industry, due to petroselinic acid content, which can be used as a plastic lubricant during the manufacturing of nylons [78].

The growing demand for improved phytochemicals extraction that are valuable to the food, pharmaceutical, agricultural and cosmetics industries, has led to the development of several extraction techniques suited to the specific compounds to be obtained. Coriander phytocompounds extraction comprise an important and growing area of research, contributing towards the valorization of this medicinal and aromatic plant.

## 4. Conclusions

This study combined three different approaches (namely ethnobotany, chemical analysis, and a literature survey) with the aim of contributing to the selection of coriander enhanced varieties to be made available for producers.

Ethnobotanical studies were essential to understand the use of coriander by the population and for which purposes, as well as which varieties are nowadays in use by producers. These results were used to publish a book with the traditional recipes, cultivation, and plant breeding results [79], to return to the people their knowledge, valuing and allowing it to be used for future generations.

The most interesting coriander features for the development of a new variety were fresh biomass yields, flavor, and late flowering. These findings were taken into account for the coriander breeding program, leading to the submission of four coriander varieties to the Portuguese National Varieties Catalogue in 2022 [30].

The chemical analysis allowed understanding how these varieties perform, in terms of essential oil and oil production. The literature survey confirmed the potential of this species, and that the optimization of extraction techniques may help meet the demands of producers and industry.

In view of the growing food, therapeutic and agricultural interest in coriander, the obtained findings may contribute to the selection of varieties with greater resistance to biotic and abiotic stress, and enhanced amounts of bioactive compounds. 

## Figures and Tables

**Table 1:** *Coriandrum sativum* accessions studied codes, BPGV accession number, collection site, plant part studied, EO and fatty acids yields.

**Sample Code**	**BPGV Accession**	**Accession Origin: Municipality/District**	**Plant Part**	**Yield (%, *v*/*v*)**
**Essential oil Analysis**				
Cs_1_API_14	BPGV08514	Elvas, Portalegre	APIs	0.04
Cs_16_API_14	BPGV19284	Vidigueira, Beja	APIs	0.05
Cs_32_API_14	BPGV28149	Campo Maior, Portalegre	APIs	0.05
Cs_TP_API_14		Commercial variety	APIs	0.04
Cs_1_F_14	BPGV08514	Elvas, Portalegre	Fruits	0.46
Cs_16_F_14	BPGV19284	Vidigueira, Beja	Fruits	0.39
Cs_26_F_13	BPGV08524	Santiago do Cacém, Setúbal	Fruits	0.25
Cs_31_F_14	BPGV19293	Campo Maior, Portalegre	Fruits	0.25
Cs_31_F_14_w	BPGV19293	Campo Maior, Portalegre	Fruits	0.14
Cs_32_F_14	BPGV28149	Campo Maior, Portalegre	Fruits	0.37
Cs_TP_F_14		Commercial variety	Fruits	0.40
**Sample Code**	**BPGV Accession**	**Accession Origin Municipality/District**	**Plant Part**	**Yield (%, *w*/*w*)**
**Fatty Acids Analysis**				
Cs_1_1_F_14	BPGV08514	Elvas, Portalegre	Fruits	0.8
Cs_1_2_F_14	BPGV08514	Elvas, Portalegre	Fruits	0.9
Cs_1_3_F_14	BPGV08514	Elvas, Portalegre	Fruits	1.2
Cs_16_1_F_14	BPGV19284	Vidigueira, Beja	Fruits	1.2
Cs_16_2_F_14	BPGV19284	Vidigueira, Beja	Fruits	0.7
Cs_16_3_F_14	BPGV19284	Vidigueira, Beja	Fruits	0.8
Cs_31_F_14_w	BPGV19293	Campo Maior, Portalegre	Fruits	0.8
Cs_32_1_F_14	BPGV28149	Campo Maior, Portalegre	Fruits	1.5
Cs_32_2_F_14	BPGV28149	Campo Maior, Portalegre	Fruits	1.1
Cs_32_3_F_14	BPGV28149	Campo Maior, Portalegre	Fruits	1.2
Cs_33_F_14_w	BPGV19649	Campo Maior, Portalegre	Fruits	0.7
Cs_Santo_1_F_14		Commercial variety	Fruits	1.0
Cs_Santo_2_F_14		Commercial variety	Fruits	0.9
Cs_Santo_3_F_14		Commercial variety	Fruits	1.5
Cs_TP_1_F_14		Commercial variety	Fruits	0.5
Cs_TP_2_F_14		Commercial variety	Fruits	0.8
Cs_TP_3_F_14		Commercial variety	Fruits	0.5

In the accession code, the two first letters stand for the species name, followed by sample number, plant part and collection year. APIs: Aerial parts with inflorescence emergence. F: Fruits. TP and Santo: Designations of commercial varieties. When the sample was obtained from wild collection the sampling year is followed by letter w. BPGV: Banco Português de Germoplasma Vegetal.

**Table 2:** Food categories from 2013 surveys in Alegrete, Santa Catarina, and Vale de Vargo, arranged by order of importance in the sum of the 3 villages.

Recipe Type	Alegrete	Santa Catarina	Vale de Vargo	Total 3 Villages
*Açorda* *	20	20	20	60
Fish	18	20	13	51
Rice	16	20	15	51
Fabaceae	11	18	17	46
Fish soup	18	19	7	44
Meat	18	11	8	37
*Piso* *	16	15	1	32
*Migas* *	2	6	0	8
Total	119	129	81	329

* *Açorda*, *migas*, and *piso*: traditional recipes detailed in Section 3.1.1.

**Table 3:** Representativeness of the food categories from the 2002–2003 and 2013 surveys recipes, arranged by order of importance in the sum of the 2 surveys.

Recipe Type		Surveys	
	2003	2013	Total
Fabaceae	19	17	36
*Açorda* * and *migas* *	24	5	29
Fish	7	19	26
Other recipes	8	16	24
Meat	4	6	10
Total	62	63	125

* *Açorda*, and *migas*: traditional recipes detailed in Section 3.1.1.

**Table 4:** Percentage composition of the EOs isolated by hydrodistillation, from *Coriandrum sativum* aerial parts with inflorescence emergence (APIs) and fruits (F). For samples codes, see Table 1.

Peak					APIs					Fruits			
#	Components	RI	Cs_1_ API_14	Cs_16_API_14	Cs_32_API_14	Cs_TP_API_14	Cs_1_F_14	Cs_16_F_14	Cs_26_F_13	Cs_31_F_14	Cs_31_F_14_w	Cs_32_F_14	Cs_TP_F_14
1	*n*-Hexanal	756					t	t	t	t	t	t	t
2	*n*-Octane	800	0.7	1.0	0.5	1.2							
3	*cis*-3-Hexen-1-ol	868	t	0.2	0.1	t							
4	*cis*-2-Hexen-1-ol	882	t	0.1	0.1	t							
5	*n*-Hexanol	882	t	0.1	t	t							
6	*n*-Heptanal	897	t	t	t	t	t	t	0.1	0.1	t	t	t
7	1-Nonene	899	t	t	t	t							
8	*n*-Nonane	900	**15.1**	**9.9**	**11.0**	**17.2**	t	t	t	0.1	t	t	t
9	*trans*-2-Nonene *	900	t	t	t	t							
10	α-Thujene	924					t	t	0.1	0.1	0.1	t	t
11	α-Pinene	930	0.5	0.4	0.4	0.6	**4.6**	**3.3**	**3.0**	**8.7**	**6.2**	**3.6**	**4.1**
12	Camphene	938					0.5	0.4	0.3	0.5	0.4	0.4	0.4
13	*n*-Heptanol	952	t	t	t	t	t	t	t	t	t	t	t
14	Sabinene	958	t	t	t	0.1	0.2	0.2	0.3	0.3	0.3	0.3	0.2
15	1-Octen-3-ol	961	t	t	t	t	t	t	t	t	t	t	t
16	β-Pinene	963	t	t	t	t	0.4	t	0.4	0.9	0.6	0.3	0.4
17	3-Methyl nonane	970	t	t	t	t							
18	2-Pentyl furan	973					t	t	0.1	t	t	t	t
19	*n*-Octanal	973	3.0	2.6	2.1	2.8	t	t	0.1	t	t	t	t
20	β-Myrcene	975					0.8	0.8	1.3	1.6	1.3	0.7	0.8
21	α-Phellandrenene	995					t	t	t	t	t	t	t
22	*n*-Decane	1000	1.2	0.6	0.7	0.8							
23	α-Terpinene	1002					t	t	0.1	0.1	0.1	t	0.1
24	*p*-Cymene	1003	t	t	t	t	0.9	1.1	1.1	0.6	1.0	1.2	1.6
25	β-Phellandrene	1005					0.1	0.1	0.1	0.1	0.2	0.1	0.1
26	Limonene	1009	t	t	t	0.1	1.4	1.4	1.4	1.6	1.3	1.2	1.4
27	*cis*-β-Ocimene	1017					t	t	0.2	0.2	0.2	t	t
28	*trans*-β-Ocimene	1027	t	t	t	t	t	t	0.3	0.3	0.3	t	t
29	γ-Terpinene	1035	t	t	t	0.1	**4.8**	**6.2**	**4.4**	**4.3**	**8.3**	**7.3**	**5.7**
30	*trans*-Sabinene hydrate	1037					t	t	t	t	t	t	t
31	*cis*-Linalool oxide	1045					t	0.3	t	0.1	t	t	0.1
32	*n*-Octanol	1045	0.4	0.3	0.3	0.4	t	t	t	t	t	t	t
33	*trans*-Linalool oxide	1059					t	t	0.1	t	t	t	0.1
34	Terpinolene	1064					0.3	t	0.2	0.3	0.3	0.3	0.3
35	*n*-Nonanal	1073	0.9	1.0	1.0	1.0							
36	Linalool	1074	0.2	0.5	0.2	0.1	**77.9**	**75.7**	**78.0**	**73.3**	**73.4**	**73.7**	**77.7**
37	1-Octen-3-yl acetate	1086	t	t	t	t							
38	*n*-Undecane	1100	0.2	t	0.1	0.1							
39	Camphor	1102					2.9	4.3	2.1	2.3	1.9	3.9	2.7
40	Citronellal	1121						t	0.1	0.6	0.9	0.1	t
41	2-*trans*-Nonenal	1124	0.1	0.2	0.2	0.1						t	
42	Borneol	1134					0.3	t	0.6	t	0.1	0.1	0.1
43	Terpinen-4-ol	1148					0.2	0.3	0.3	0.2	0.2	0.4	0.3
44	*n*-Nonanol	1148	0.3	0.5	0.4	0.3							
45	α-Terpineol	1159					0.3	0.4	0.3	0.2	0.2	0.3	0.3
46	4-*cis*-Decenal	1163	0.2	0.3	0.2	0.2							
47	*n*-Decanal	1180	**23.0**	**20.9**	**21.0**	**24.1**	0.1	0.1	0.4	0.5	0.3	0.2	0.2
48	Pulegone	1207	t	t	t	t	0.1	t	0.1	0.1	t	t	0.1
49	Citronellol	1207	t	t	t	t	0.1	t	0.1	0.5	0.6	0.1	t
50	Geraniol	1236					2.0	3.0	2.0	0.8	0.6	3.3	1.4
51	2-*trans*-Decenal	1236	**12.2**	**17.9**	**16.1**	**13.1**							
52	2-*trans*-Decen-1-ol *	1256	**9.2**	**9.8**	**8.8**	**7.5**							
53	*n*-Decanol	1259	6.7	6.5	6.0	7.1	t	t	0.1	0.1	0.1	t	t
54	*n*-Undecanal	1288	4.0	4.2	4.2	3.2							
55	Myrtenyl acetate	1290					t	t	0.1	0.1	t	0.1	t
56	2-*trans*-Undecenal	1323	1.9	3.7	3.2	1.6							
57	2-*trans*-Undecen-1-ol *	1325	0.7	1.2	1.0	0.5							
58	Citronellyl acetate	1343					t	t	t	0.1	t	t	t
59	Neryl acetate	1353					t	t	t	t	t	t	t
60	*n*-Undecanol	1366	0.5	0.6	0.5	0.4							
61	Geranyl acetate	1370					1.3	1.1	1.8	1.0	0.8	0.8	1.6
62	*n*-Dodecanal	1397	4.3	3.2	3.8	3.8	t	t	0.1	t	t	t	t
63	2-*trans*-Dodecenal	1446	5.3	5.4	5.3	5.1	0.3	t	0.2	0.2	0.2	0.2	t
64	2-*trans*-Dodecen-1-ol	1448	1.1	1.2	1.1	1.0							
65	*n*-Dodecanol	1468	0.2	0.2	0.2	0.2							
66	α-Amorphene	1476	0.1	t	t	t							
67	*n*-Tridecanal	1499	0.4	0.3	0.3	0.4							
68	2-*trans*-Tridecen-1-al	1574	0.4	0.2	0.4	0.4							
69	*n*-Tetradecanal (=miristaldehyde)	1596	0.7	0.6	0.9	0.7							
70	α-Cadinol	1626	0.1	t	t	t							
71	*cis*-9-Tetradecenal *	1647	2.9	2.7	3.4	2.9							
72	*n*-Pentadecanal	1688	0.1	0.1	0.1	0.1							
73	11-Pentadecenal *	1735	0.5	0.4	0.4	0.5							
74	Hexadecanoic acid (=palmitic acid)	1908	t	t	t	t							
75	Phytol acetate 2	2101	0.1	t	t	t							
76	Methyl linoleate	2101	0.1	t	t	t							
77	*n*-Nonadecanal *	2102	t	t	t	t							
78	Petroselinic acid	2128					t	t	t	t	t	t	t
79	*n*-Eicosanal	2200	0.1	t	0.1	t							
	**% Identification**		97.4	96.8	94.1	97.7	99.5	98.7	99.9	99.9	99.9	98.6	99.7
	**Grouped components**												
	Monoterpene hydrocarbons		0.5	0.4	0.4	0.9	14.0	13.5	13.2	19.6	20.6	15.4	15.1
	Oxygen-containing monoterpenes		0.2	0.5	0.2	0.1	85.1	85.1	85.6	79.3	78.7	82.8	84.4
	Sesquiterpene hydrocarbons		0.1	t	t	t							
	Oxygen-containing sesquiterpenes		0.1	t	t	t							
	Oxygen-containing diterpenes		0.1	t	t	t							
	Fatty acids		t	t	t	t	t	t	t	t	t	t	t
	Fatty acid derivatives		79.2	84.0	81.0	77.4	0.4	0.1	1.0	0.9	0.6	0.4	0.2
	Others		17.2	11.7	12.4	19.3	t	t	0.1	0.1	t	t	t

Peak #: Peak number. RI: In-lab calculated retention index relative to C_8_-C_23_ *n*-alkanes on the DB-1 column. t: traces (t < 0.05%). * Identification based on mass spectrum only. Percentages in bold: dominant compounds relevant for each cluster.

**Table 5:** Percentage composition of the fatty acids isolated using a Soxhlet apparatus, from *Coriandrum sativum* fruits (F), after transesterification to fatty acid methyl esters (FAMES). For samples codes, see Table 1.

Peak #	FAMES	Fatty Acids	Formula	*C:D*	ω	Cs_1_F_14	Cs_16_F_14	Cs_31_F_14_w	Cs_32_F_14	Cs_33_F_14_w	Cs_ Santo_F_14	Cs_ TP_ F_14
1	Methyl dodecanoate (=Methyl laurate)	Dodecanoic acid (=lauric acid)	C_12_H_24_O_2_	C12:0		t	t	t	t	t	t	t
2	Methyl tridecanoate	Tridecanoic acid (=tridecylic acid)	C_13_H_26_O_2_	C13:0		t	t	t	t	t	t	t
3	Methyl tetradecanoate (=methyl myristate)	Tetradecanoic acid (=myristic acid)	C_14_H_28_O_2_	C14:0		7.6	17.5	6.5	4.2	4.1	5.6	4.9
4	Methyl pentadecanoate	Pentadecanoic acid (=pentadecylic acid)	C_15_H_30_O_2_	C15:0		t	t	t	t	t	t	t
5	*cis*-10-Heptadecenoic acid methyl ester	*cis*-10-Heptadecenoic acid	C_17_H_32_O_2_	C17:1 *cis*-10	ω 7	t	t	t	t	t	t	t
6	Methyl *cis*-9-hexadecenoate (=methyl palmitoleate)	*cis*-9-Hexadecenoic acid (=palmitoleic acid)	C_16_H_30_O_2_	C16:1 *cis*-9	ω 7	t	t	t	t	t	t	t
7	Methyl hexadecanoate (=methyl palmitate)	Hexadecanoic acid (=palmitic acid)	C_16_H_32_O_2_	C16:0		19.2	29.7	27.3	12.9	22.3	14.7	12.0
8	Methyl heptadecanoate (=methyl margarate)	Heptadecanoic acid (=margaric acid)	C_17_H_34_O_2_	C17:0		t	t	t	t	t	t	t
9	Methyl *cis*,*cis*-9,12-octadecadienoate (=methyl linoleate)	(9Z,12Z)-9,12-Octadecadienoic acid (=linoleic acid, grape seed oil)	C_18_H_32_O_2_	C18:2 *cis*-9, *cis*-12	ω 6	16.7	14.0	7.6	19.1	12.3	18.3	19.2
10	Methyl *cis*-6-octadecenoate (=methyl petroselinate)	(6Z)-Octadec-6-enoic acid (=petroselinic acid)	C_18_H_34_O_2_	C18:1 *cis*-6	ω 12	45.6	31.5	45.1	55.3	54.1	54.7	55.4
11	Methyl *cis*-9-octadecenoate (=methyl oleate)	(9Z)-Octadec-9-enoic acid (=oleic acid)	C_18_H_34_O_2_	C18:1 *cis*-9	ω 9	t	t	t	t	t	t	t
12	Methyl octadecanoate (=methyl stearate)	Octadecanoic acid (=stearic acid)	C_18_H_36_O_2_	C18:0		3.9	4.6	8.1	2.7	1.7	2.8	2.1
13	Methyl eicosanoate (=methyl arachidate)	Eicosanoic acid (=arachidic acid)	C_20_H_40_O_2_	C20:0		t	t	t	t	t	t	t
		**% of identification**				93.1	97.3	94.6	94.2	94.5	96.2	93.6
	**Fatty acids ratio**											
	Petroselinic acid:Myristic acid		(C18:1 *cis*-6):(C14:0)			6.1	1.8	6.9	13.1	13.2	9.8	11.6
	Petroselinic acid:Palmitic acid		(C18:1 *cis*-6):(C16:0)			2.4	1.1	1.7	4.3	2.4	3.8	4.8
	Petroselinic acid:Linoleic acid		(C18:1 *cis*-6):(C18:2)			2.7	2.3	5.9	2.9	4.4	3.0	2.9
	Petroselinic acid:Stearic acid		(C18:1 *cis*-6):(C18:0)			2.7	2.3	5.9	2.9	4.4	3.0	2.9
	**Average extract yield (%, *w*/*w*)**					1.0	0.9	0.8	1.3	0.7	1.1	0.6

Peak #: Peak number. C:D: where C is the number of carbon atoms in the fatty acid and D is the number of double bonds in the fatty acid. ω: Number of carbons from the methyl end to the first carbon in the double bond of the fatty acid, according to Omega nomenclature. t: traces (t < 1%).

## Data Availability

The original contributions presented in the study are included in the article/Supplementary Material, further inquiries can be directed to the corresponding author.

## Supplementary Materials

The following supporting information can be downloaded at: https://www.mdpi.com/article/10.3390/foods13060929/s1, Table S1. Survey questionnaire used during 2003 in 28 coriander landraces origins across Alentejo region (see Figure 1). Table S2. Survey questionnaire used during 2013 in the villages of Alegrete, Santa Catarina and Vale de Vargo (see Figure 1). Table S3. Survey questionnaire used during 2007 in Beja, Elvas and Évora indoor markets and Beja and Estremoz outdoor markets. Table S4. Survey questionnaire used during 2022 in the markets of Elvas, Estremoz and Portalegre (see Figure 1). Figure S1. *Coriandrum sativum* vegetative aerial parts with inflorescence emergence (A) and fruits, with detail of the field trial with plant material at full ripening stage (B,C). Figure S2. Dendrogram obtained by cluster analysis of the EOs from *C. sativum* vegetative aerial parts and fruits, based on correlation and using the unweighted pair group method with the arithmetic average. For the samples’ codes in each of cluster I and II, see Table 1. Figure S3. Representative gas chromatography profiles of the EOs from *C. sativum* vegetative aerial parts and fruits. Figure S4. Dendrogram obtained by cluster analysis of the fatty acids from *C. sativum* fruits, based on correlation and using the unweighted pair group method with the arithmetic average. For the samples’ codes, see Table 1. Figure S5. Chromatographic profile of the fatty acid from coriander fruits, analyzed in the form of FAMES.
