# Supplementary material for: Coriander (Coriandrum sativum L.) from Alentejo (South Portugal)—Ethnobotany and Potential Industrial Use
Source: Foods. 2024 Mar 19;13(6):929. doi: 10.3390/foods13060929 (PMC10969941; doi:10.3390/foods13060929)
Supplement: Supplementary file 1 [file foods-13-00929-s001.zip › foods-2872601-supplementary.pdf]

S2. Materials and Methods

S2.1. Ethnobotanical surveys: food seasoning with coriander in Alentejo region

S2.1.1. Surveys Conducted in 2002-2003

Table S1. Survey questionnaire used during 2002-2003 in 28 coriander landraces origins across Alentejo region (see Figure 1).

**Survey date**
**Survey code** *
**1. Informant characterization**
1.1 Gender (Man/Woman)
1.2 Age (number)
1.3 School instruction
**2. Coriander food seasoning utilization frequency**
> 1 time/week
> 1 time/month
1 time/ month
< 1 time/ month
**3. Coriander medicinal utilization**
Respiratory system problems
Digestive system problems
Circulatory system problems
Other
**4. Coriander food seasoning recipes**
Açorda and *migas* **
Rice
Meat
Fabaceae (beans, chickpea, pea)
Omelette
Liquor
Potatoes
Fish
Salad
Soup
Fish soup
Piso ** food sauce
Other
**Field notes**

* Numeric code (Cs1 to Cs 28) attributed by chronologic order, considering survey date. ** Traditional recipes explained in sub-section 3.1.1. from the results and discussion section.

S2.1.2. Surveys Conducted in 2013

Table S2. Survey questionnaire used during 2013 in the villages of Alegrete, Santa Catarina and Vale de Vargo (see Figure 1).

Survey date
Survey code *
1. Informant
- 1.1 Gender (Man/Woman)
- 1.2 Age (number)
- 1.3 School instruction

2. Coriander seed origin
- Landrace
- Commercial seed

3. Coriander seed propagation
- Acquired
- From own cultivation
- Neighbour offer

4. Coriander food seasoning use frequency
- > 1time/week
- > 1time/month
- 1 time/ month
- < 1time/ month

5. Medicinal use
- Respiratory problems
- Digestive problems
- Oher

6. Coriander food seasoning recipes categories
(cross marking)
- *Açorda* and *migas* **
- Rice
- Meat
- Fabaceae (beans, chickpea, pea)
- Omelette
- Liquor
- Potatoes
- Fish
- Salad
- Soup
- Fish soup
- *Piso* **
- Other

7. Recipes of food with coriander (text)
Field notes

* Numeric code attributed by chronologic order, considering survey date; Alegrete (Al) 1 to 24, Santa Catarina (SC) 1 to 20, Vale de Vargo (VV) 1 to 21. ** Traditional recipes explained in sub-section 3.1.1. from the results and discussion section.

S2.2. Ethnobotanical surveys: coriander representativeness on local markets

S2.2.1. Surveys Conducted in 2007

Table S3. Survey questionnaire used during 2007 in Beja, Elvas and Évora indoor markets and Beja and Estremoz outdoor markets.

Survey date
Survey code *
1. Producer characterization
- 1.1 Gender (Man/Woman)
- 1.2 Age (number)
- 1.3 Scholl instruction

2. Cultivated plant identification **
- 2.1 Cultivated plant name
- 2.2 Cultivated plant area (m^2)
- 2.3 MAP (number of species)
- 2.4 MAP (total area, m^2)

3. Coriander (*Coriandrum sativum*)
- 3.1 Sowings/year (number)
- 3.2 Cuts/year (number)
- 3.3 Cuts/sowing date (number)
- 3.4 Seed origin
  - Landrace
  - Acquired
- 3.5 Landrace conservation is important
  - Yes
  - No
- 3.6 Develop new variety is important
  - Yes
  - No
- 3.7 Features of new variety
  - Yield: vegetative biomass
  - Flavor
  - Crop resistance (diseases and insects)
  - Late flowering
  - Larger fridge post-harvest conservation
  - External look
  - Fast growing
  - Larger leaf's

Field notes

* Numeric code attributed by chronologic order, considering survey date. Beja indoor market (Bi) 1 to 8. Beja outdoor market (Bo) 1 to 9. Évora indoor market (Ei) 1 to 4. Estremoz outdoor market (Eo) 1 to 10 and Elvas indoor market (Ei) 1 to 2. MAP: Medicinal and aromatic plant ** repeat as many times as necessary for each MAP producer.

S2.2.2. Surveys Conducted in 2022

Table S4. Survey questionnaire used during 2022 in the markets of Elvas, Estremoz and Portalegre (see Figure 1).

**Survey date**<br>**Survey code***<br>**MAP Plant name********	**Origin of the plant****	**Packaging*****	**Field notes**

* Elvas (El) 1 to 4. Portalegre (Po) 1 to 11. Estremoz (Es) 1 to 13. **Origin of the plant: cultivated or wild (collected in the nature). ***Packaging: bulk, plastic bag, other (plastic paper, container, net bag). MAP: Medicinal and aromatic plant ****repeat as many times as necessary for each MAP producer.

S2.3. Essential Oils Isolation and Analysis

The essential oils (EOs) were isolated by hydrodistillation (HD), for 3 h at a distillation rate of 3 mL/min, using a Clevenger apparatus, according to the European Pharmacopoeia [36]. *Coriandrum sativum* accessions studied are detailed in Table 1 and representative examples depicted in Figure S1.

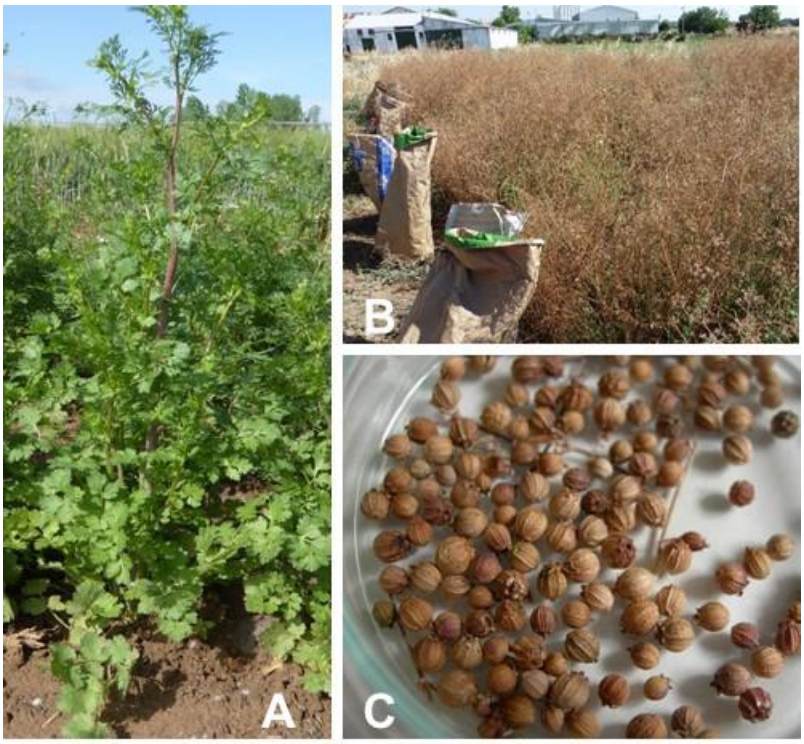

Figure S1. *Coriandrum sativum* vegetative aerial parts with inflorescence emergence (A) and fruits, with detail of the field trial with plant material at full ripening stage (B-C).

The EOs were analysed by Gas Chromatography (GC) and Gas Chromatography-Mass Spectrometry (GC-MS) for component quantification and identification, respectively.

GC analyses were run either on a Clarus 400 or a Perkin-Elmer AutoSystem 9000 gas chromatograph (Perkin-Elmer, Shelton, CT, USA), each of which was equipped with two flame ionization detectors, with a data handling system and a split-splitless injector port into which two columns of different polarities were inserted: a DB-1 fused-silica column (polydimethylsiloxane, 30 m x 0.25 mm i.d., film thickness 0.25 μm; J & W Scientific Inc., Rancho Cordova, CA, USA) and a DB-17HT fused-silica column [(50% phenyl)-methylpolysiloxane, 30 m x 0.25 mm i.d., film thickness 0.15 μm; J & W Scientific Inc.]. The oven temperature was programmed at 3 °C/min from 45 to 175 °C, then at 15 °C/min

up to 300 °C, and finally held isothermal for 10 min. The temperatures of the injector and detector were 280 °C and 300 °C, respectively. The carrier gas was hydrogen (30 cm/s). The split sampling technique ratio was 1:50. The injection volume was 0.1 µL of a 1:1 distilled *n*-pentane-essential oil solution. The EOs percentage composition was determined using the normalization method for the GC peak areas, calculated as the average of two injections per sample, without using the response factors, according to ISO 7609 [37].

GC-MS analyses were performed on a Perkin Elmer Clarus 600 gas chromatograph, equipped with a DB-1 fused-silica column as described above, and interfaced with a Perkin-Elmer 600T mass spectrometer (software version 5.4.2.1617, Perkin Elmer, Shelton, CT, USA). Oven and injector temperatures were the same as for GC analyses. Transfer line at 280 °C. Carrier gas, helium (30 cm/s). Ion source at 220 °C. Split ratio, 1:40. Ionization energy, 70 eV. Scan range at 40-300 u and scan time of 1 s. Components identity was established by comparison of their retention indices, calculated according to ISO 7609 [38], relative to *n*-alkane (Sigma) indices and GC-MS spectra from a laboratory library generated with commercially available standards (Extrasynthese, Cymit Química, S.L.; Sigma-Aldrich; Fluka, Riedel-de Haën), laboratory-synthesized components [38,39] laboratory isolated compounds [40-44], and reference essential oils of *Thymus caespititius* [45], *Juniperus cedrus* {46] and *Cryptomeria japonica* [47] in which the components' identity was confirmed by RI, GC-MS, and ^{13}C-NMR, according to in-lab procedures [48].

S2.4. Fatty Acids Isolation and Analysis

For fatty acid isolation, coriander fruits (≈ 10g d.w.) were loaded in a hand-made Whatman filter paper extraction thimble, which was placed in the main chamber of the Soxhlet extractor and extracted, for 3 h, using *n*-hexane as the extraction solvent. The resulting extracts were concentrated to dryness at 45 °C under reduced pressure on a rotary evaporator (Yamato, Hitec RE-51), for yield determination. Each extract was then dissolved in 500 µl of *n*-hexane and stored at -20 °C until analysis.

Acid-catalyzed transesterification was used to obtain the fatty acid methyl esters (FAMES). The reaction mixture consisted of 20 µl of extract, 2 ml anhydrous methanolic HCl 2 N (prepared as detailed below), and 0.5 ml of *n*-hexane. The reaction mixtures were transferred to glass vials, capped with polytetrafluoroethylene (PTFE) stoppers with PTFE cap liners. After homogenizing, the reaction mixture was left to react at 90 °C for 90 min in a dry bath incubator. At the end of the reaction and after cooling to room temperature, 5 ml of ultrapure water and 0.5 ml of *n*-hexane were added. The vial was then vortexed for 1 min and allowed to stand, for 1 min, until layer separation was complete. The upper hexane plus lipid layer was then transferred to a clean vial and concentrated to ≈ 30 µl, using a blow-down evaporator system, at room temperature under nitrogen flow. Each mixture was stored in the dark at -20°C until analysis.

Fresh anhydrous methanolic HCl was prepared in a fume hood, by adding dropwise 0.8 ml acyl chloride to 5 ml anhydrous methanol while stirring, in a cold bath. The mixture was left to stand at room temperature for 5 min and then used.

FAMES were analysed by GC and GC-MS for component quantification and identification, respectively, using commercially available standards as detailed is section 2.3, namely individual standards, and F.A.M.E. Mix, C4-C24, form Sigma, following in-lab procedures.

GC instrumentation was as described above. Injections were made in split mode (1:50). The oven temperature was kept isothermal at 170 °C for 3 min, and then programmed at 5 °C/min up to 270 °C, and finally held isothermal for 5 min. The temperatures of the injector and detector were 290 °C and 300 °C, respectively. The carrier gas was hydrogen (30 cm/s). The percentage composition of FAMES was determined using the normalization method from the GC peak areas, calculated as an average of two injections per sample, without using the response factors.

The GC-MS instrument, oven temperature, operating conditions (except for the scan range which was 40-350 u), and components identification procedure were as described for EOs analysis.

S3. Results and Discussion

S3.3. Coriander Aerial Parts and Fruits Essential Oils

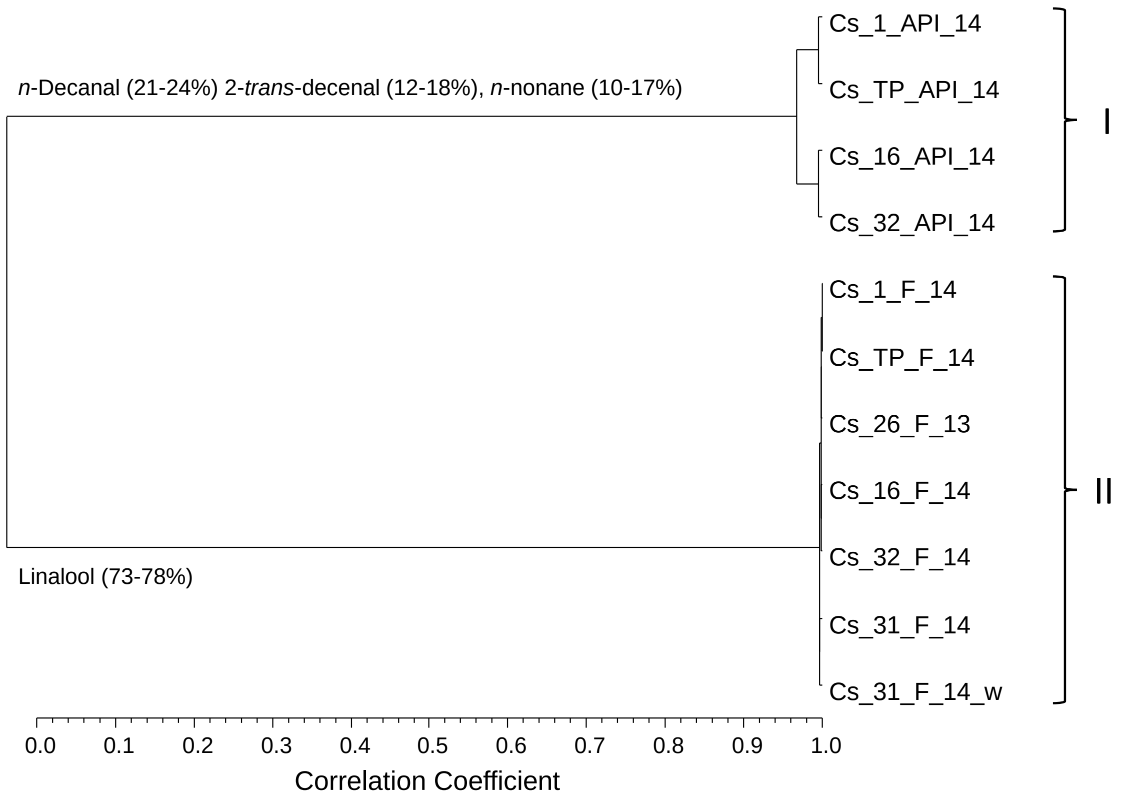

Figure S2. Dendrogram obtained by cluster analysis of the EOs from *C. sativum* vegetative aerial parts and fruits, based on correlation and using the unweighted pair group method with the arithmetic average. For the samples' codes in each of cluster I and II, see Table 1.

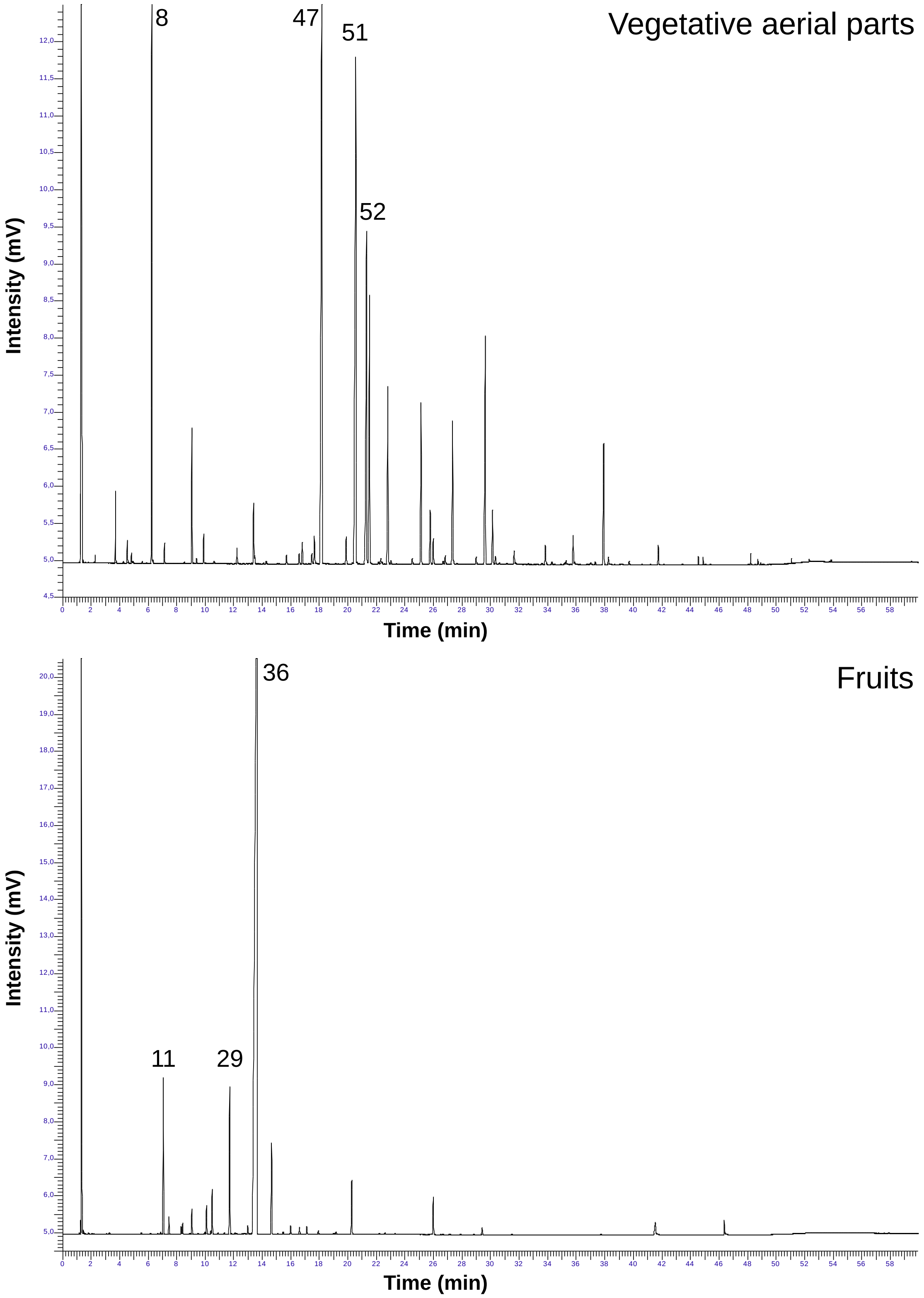

Figure S3. Representative gas chromatography profiles of the EOs from *C. sativum* vegetative aerial parts and fruits. For peak names see Table 4.

3.4. Coriander Fruits Fatty Acids

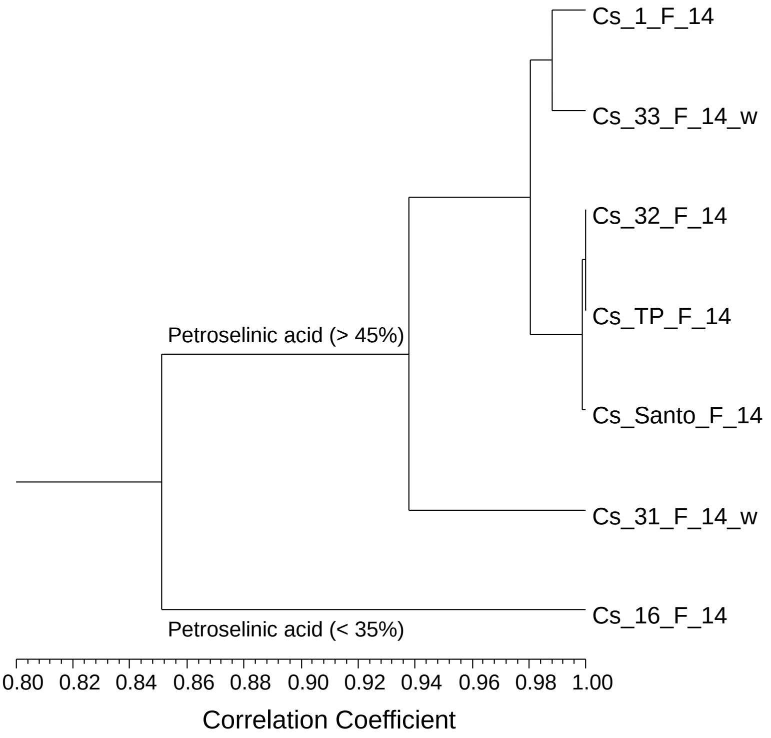

Figure S4. Dendrogram obtained by cluster analysis of the fatty acids from *C. sativum* fruits, based on correlation and using the unweighted pair group method with the arithmetic average. For the samples' codes, see Table 1.

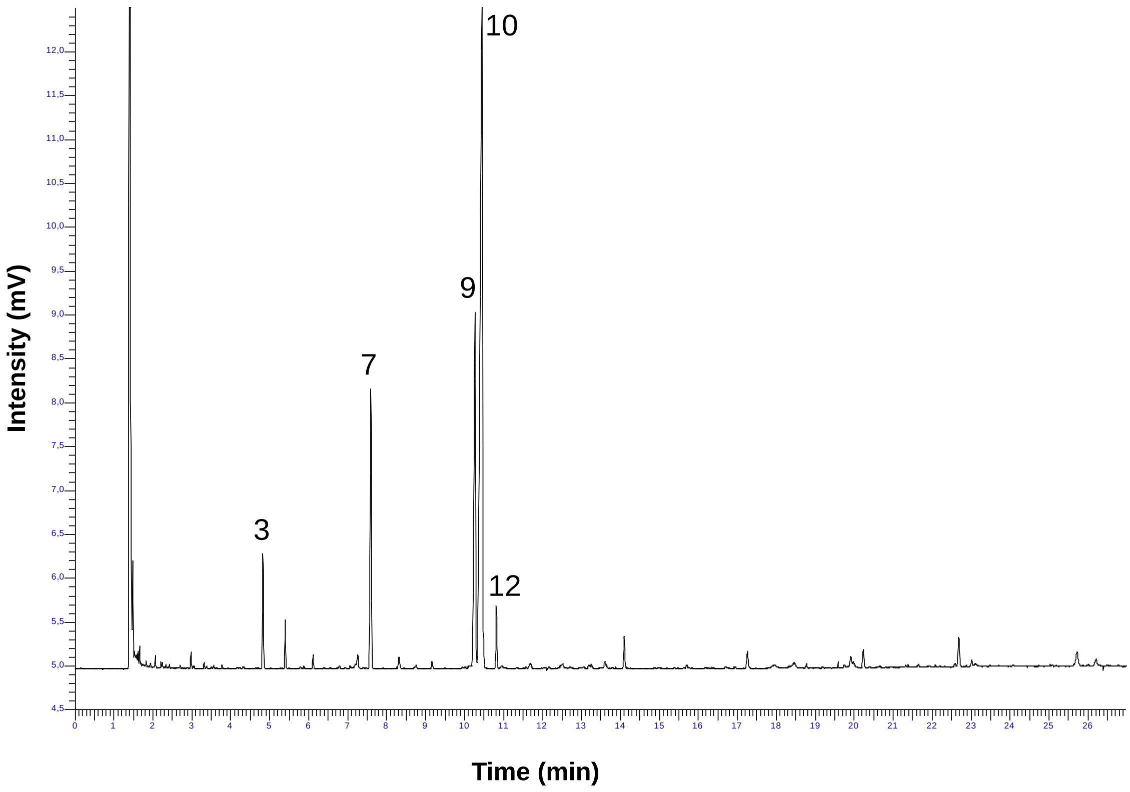

Figure S5. Chromatographic profile of the fatty acid from coriander fruits, analyzed in the form of FAMES. For peak names see Table 5.

References

36. Council of Europe; European Pharmacopoeia Commission; European Directorate for the Quality of Medicines & Healthcare. *European Directorate for the Quality of Medicines*, in *European Pharmacopoeia*, 7th ed.; Council of Europe, European Directorate for the Quality of Medicines and Healthcare: Strasbourg, France, 2010; p. 241.
37. ISO 7609:1985; Essential Oils-Analysis by Gas Chromatography on Capillary Columns-General Method. ISO: Geneva, Switzerland, 1985.
38. Santos, P.M.; Figueiredo, A.C.; Oliveira, M.M.; Barroso, J.G.; Pedro, L.G.; Deans, S.G.; Younus, A.K.M.; Scheffer, J.J.C. Essential Oils from Hairy Root Cultures and from Fruits and Roots of *Pimpinella anisum*. *Phytochemistry* **1998**, *48*, 455–460.
39. Figueiredo, A.C.; Sim-Sim, M.; Barroso, J.G.; Pedro, L.G.; Santos, P.A.G.; Fontinha, S.S.; Schripsema, J.; Deans, S.G.; Scheffer, J.J.C. Composition of the Essential Oil From the Liverwort *Marchesinia mackaii* (Hook.) S. F. Gray Grown in Portugal. *J. Essent. Oil Res.* **2002**, *14*, 439–442.
40. Figueiredo, A.C.; Barroso, J.G.; Pais, M.S.S.; Scheffer, J.J.C. Composition of the Essential Oils from Leaves and Flowers of *Achillea millefolium* L. ssp. *millefolium*. *Flavour Fragr. J.* **1992**, *7*, 219–222.
41. Ascensão, L.; Figueiredo, A.C.; Barroso, J.G.; Pedro, L.G.; Schripsema, J.; Deans, S.G.; Scheffer, J.J.C. *Plectranthus madagascariensis*: Morphology of the Glandular Trichomes, Essential Oil Composition, and Its Biological Activity. *Int. J. Plant Sci.* **1998**, *159*, 31–38.
42. Santos, P.A.G.; Figueiredo, A.C.; Lourenço, P.M.L.; Barroso, J.G.; Pedro, L.G.; Oliveira, M.M.; Schripsema, J.; Deans, S.G.; Scheffer, J.J.C. Hairy Root Cultures of *Anethum graveolens* (dill): Establishment, Growth, Time-Course Study of Their Essential Oil and Its Comparison with Parent Plant Oils. *Biotechnol. Lett.* **2002**, *24*, 1031–1036.
43. Faria, J.M.S.; Sena, I.; Moiteiro, C.; Bennett, R.N.; Mota, M.; Figueiredo, A.C. Nematotoxic and Phytotoxic Activity of *Satureja montana* and *Ruta graveolens* Essential Oils on *Pinus pinaster* Shoot Cultures and *P. pinaster* with *Bursaphelenchus xylophilus* in vitro Co-cultures. *Ind. Crops Prod.* **2015**, *77*, 59–65.
44. Faria, J.M.S.; Rodrigues, A.M.; Sena, I.; Moiteiro, C.; Bennett, R.N.; Mota, M.; Figueiredo, A.C. Bioactivity of *Ruta graveolens* and *Satureja montana* Essential Oils on *Solanum tuberosum* Hairy Roots and *Solanum tuberosum* Hairy Roots with *Meloidogyne chitwoodi* Co-cultures. *J. Agric. Food Chem.* **2016**, *64*, 7452–7458.
45. Salgueiro, L.R.; Vila, R.; Tomi, F.; Figueiredo, A.C.; Barroso, J.G.; Cañigueral, S.; Casanova, J.; Proença da Cunha, A.; Adzet, T. Variability of Essential Oils of *Thymus caespititius* from Portugal. *Phytochemistry* **1997**, *45*, 307–311.
46. Cavaleiro, C.; Salgueiro, L.; Barroso, J.G.; Figueiredo, A.C.; Pedro, L.G.; Fontinha, S.S.; Bighelli, A.; Casanova, J.; Looman, A.; Scheffer, J.J.C. Composition of the Essential Oil of *Juniperus cedrus* Webb & Berth. Grown on Madeira. *Flavour Fragr. J.* **2002**, *17*, 111–114.
47. Figueiredo, A.C.; Moiteiro, C.; Rodrigues, M.C.S.M.; Almeida, A.J.R.M. Essential Oil Composition from *Cryptomeria japonica* D.Don Grown in Azores: Biomass Valorization from Forest Management. *Nat. Prod. Commun.* **2021**, *16*, 1934578X211038431
48. Machado A. M., V. Lopes, A. M. Barata, O. Póvoa, N. Farinha, A. C. Figueiredo Chemical Variability of the Essential Oils from Two Portuguese Apiaceae: *Coriandrum sativum* L. and *Foeniculum vulgare* Mill. *Plants* (Basel) 2023. 12: 2749. https://doi.org/10.3390/plants12142749.
